# Association between resting-state connectivity patterns in the defensive system network and treatment response in spider phobia—a replication approach

**DOI:** 10.1038/s41398-024-02799-x

**Published:** 2024-03-07

**Authors:** Elisabeth J. Leehr, Fabian R. Seeger, Joscha Böhnlein, Bettina Gathmann, Thomas Straube, Kati Roesmann, Markus Junghöfer, Hanna Schwarzmeier, Niklas Siminski, Martin J. Herrmann, Till Langhammer, Janik Goltermann, Dominik Grotegerd, Susanne Meinert, Nils R. Winter, Udo Dannlowski, Ulrike Lueken

**Affiliations:** 1 Institute for Translational Psychiatry, University of Münster, Münster, Germany; 2 Department of Psychiatry, Psychosomatics, and Psychotherapy, Center for Mental Health, University Hospital of Würzburg, Würzburg, Germany; 3 Department of General Psychiatry, University of Heidelberg, Heidelberg, Germany; 4 Institute of Medical Psychology and Systems Neuroscience, University of Münster, Münster, Germany; 5 Otto-Creutzfeld Center for Cognitive and Behavioral Neuroscience, University of Münster, Münster, Germany; 6 Institute for Clinical Psychology and Psychotherapy, University of Siegen, Siegen, Germany; 7 Institute for Biomagnetism and Biosignalanalysis, University of Münster, Münster, Germany; 8 Institute of Psychology, Unit of Clinical Psychology and Psychotherapy in Childhood and Adolescence, University of Osnabrück, Osnabrück, Germany; 9 Department of Psychology, Humboldt-Universität zu Berlin, Berlin, Germany; 10 German Center for Mental Health (DZPG), partner site Berlin/Potsdam, Berlin, Germany

**Keywords:** Predictive markers, Biomarkers

## Abstract

Although highly effective on average, exposure-based treatments do not work equally well for all patients with anxiety disorders. The identification of pre-treatment response-predicting patient characteristics may enable patient stratification. Preliminary research highlights the relevance of inhibitory fronto-limbic networks as such. We aimed to identify pre-treatment neural signatures differing between exposure treatment responders and non-responders in spider phobia and to validate results through rigorous replication. Data of a bi-centric intervention study comprised clinical phenotyping and pre-treatment resting-state functional connectivity (rsFC) data of *n* = 79 patients with spider phobia (discovery sample) and *n* = 69 patients (replication sample). RsFC data analyses were accomplished using the Matlab-based CONN-toolbox with harmonized analyses protocols at both sites. Treatment response was defined by a reduction of >30% symptom severity from pre- to post-treatment (Spider Phobia Questionnaire Score, primary outcome). Secondary outcome was defined by a reduction of >50% in a Behavioral Avoidance Test (BAT). Mean within-session fear reduction functioned as a process measure for exposure. Compared to non-responders and pre-treatment, results in the discovery sample seemed to indicate that responders exhibited stronger negative connectivity between frontal and limbic structures and were characterized by heightened connectivity between the amygdala and ventral visual pathway regions. Patients exhibiting high within-session fear reduction showed stronger excitatory connectivity within the prefrontal cortex than patients with low within-session fear reduction. Whereas these results could be replicated by another team using the same data (cross-team replication), cross-site replication of the discovery sample findings in the independent replication sample was unsuccessful. Results seem to support negative fronto-limbic connectivity as promising ingredient to enhance response rates in specific phobia but lack sufficient replication. Further research is needed to obtain a valid basis for clinical decision-making and the development of individually tailored treatment options. Notably, future studies should regularly include replication approaches in their protocols.

## Introduction

Even though exposure-based cognitive behavioral therapy (CBT) provides a powerful approach for the treatment of anxiety disorders, a substantial proportion of patients does not respond in a clinically meaningful way [1, 2]. The combination of high prevalence of anxiety disorders [3] with high rates of non-responders and disorder-related high socio-economic costs [4] urges clinical research to identify pre-treatment patient characteristics associated with treatment response. Knowledge about these characteristics may enable patient stratification, the personalized and tailored application of modified or add-on treatments and thus the improvement of response rates.

Exposure-based CBT owes its efficacy to the extensive scientific efforts that have been made with respect to the underlying neurobiology of anxiety disorders [5] and the way how fear extinction alters those neural substrates [6, 7]. In line with calls for more translationally oriented clinical research [8–10], the present investigation aims to identify pre-treatment neural signatures of treatment response.

To investigate neural markers for treatment response, we selected spider phobia as a prototypical model of fear circuitry dysfunctions in anxiety disorders. Its neurocircuitry substantially overlaps with structures commonly referred to the defensive system network within the whole spectrum of anxiety disorders [5]. Furthermore, this neurocircuitry highly corresponds with the one involved in fear extinction, which is discussed as a core mechanism of action underlying exposure treatment [7]. A network for defensive mobilization comprising the amygdala, medial prefrontal cortex (MPFC), anterior cingulate cortex (ACC), insula, and thalamus has been shown to be hyperresponsive in specific phobia patients compared to healthy controls [11–19]. This hyperactivation is accompanied by decreased activation in medial and ventral portions of the prefrontal cortex [11, 14, 20–22] thus underlying emotion regulation deficits, including deficient top-down fear inhibition [11]. Regarding the animal subtype of specific phobia, there is especially high consistency within the results [11, 18].

Neurofunctional studies on pre-treatment neural signatures in anxiety disorders are accumulating within the last years [23–29] and hint at potential moderators of treatment outcome in anxiety disorders [24, 30, 31]. Recent reviews and meta-analyses identified activation differences in regions of salience and interoceptive processing, namely the right inferior gyrus and the anterior insular cortex, as well as the dorsomedial prefrontal cortex and the dorsal anterior cingulate cortex as relevant predictors for treatment outcome in anxiety disorders [24, 26, 31]. Additionally, one meta-analysis found evidence for an activation decrease from pre- to post-treatment in symptom provocation paradigms in the left anterior cingulate, the bilateral middle frontal gyrus and the right insula [32]. However, there is little knowledge on functional connections between the mentioned neural structures.

The few existing studies on (functional) connectivity in specific phobia point to a decoupling of prefrontal structures and the defensive system during stimulus presentation [33, 34]. This was also demonstrated for other anxiety disorders [35–42]. Stefanescu et al. [34] interpreted the decoupling in terms of deficient emotion regulation. Corroborating these results, Böhnlein et al. [43] found a hypoconnectivity of the amygdala within regions involved in the salience network in patients with spider phobia compared to healthy controls during disorder-unspecific emotion processing. Furthermore, Scharmüller et al. [44] have demonstrated that fronto-striatal connectivity is decreased in specific phobia compared to healthy controls suggesting an altered information flow in those patients. However, all studies used task-based approaches, leaving open the question as to which extent these alterations may represent overarching signatures. Intrinsic brain connectivity investigated via fMRI in a resting condition is an easy-to-acquire and paradigm-independent measure. The moderating role of resting-state functional connectivity (rsFC) with respect to treatment response in specific phobia patients remains unknown. Analyzing rsFC may therefore constitute an innovative approach to better understand large-scale intrinsic brain networks conferring treatment response towards exposure treatment in spider phobia.

The present investigation aims at identifying pre-treatment rsFC signatures that differ between exposure treatment responders and non-responders in spider phobia. To contribute to the conquest of the so-called ‘replication crisis’ [45, 46] we applied a rigorous replication design by a) trying to replicate results by another team using the same data (cross-team replication) and b) trying to replicate discovery sample results in the independent replication sample (cross-site replication).

We hypothesized (1) that treatment responders exhibit enhanced negative connectivity between prefrontal areas and defensive-system networks compared to non-responders. This should be pronounced in the amygdala and the ACC. (2) We assumed that comprehensive cross-team replication is possible in the case of harmonized analysis protocols. Cross-site replication would strengthen the validity of the results.

## Methods

### Study design and sample description

Analyses are part of the “SpiderVR” study, which was conducted as a bicentric study in Würzburg and Münster within the German Collaborative Research Center 58 “Fear, Anxiety, Anxiety Disorders”.

Spider phobia patients were recruited at both sites. Fulfillment of DSM-IV-TR diagnostic criteria for specific phobia, animal subtype was assessed via the structured clinical interview for DSM-IV-TR (SCID Axis I; German version [47]). Patients had to be right-handed, of Caucasian descent, without an acute or lifetime diagnosis of comorbid mental disorders except for mild to moderate depression (unless currently treated psychotherapeutically or pharmacologically) and further specific phobias of the animal subtype. A current pregnancy or fulfillment of MRI-contraindications led to exclusion. All patients needed to reach a minimum Spider Phobia Questionnaire (SPQ, [48]) score of 20 to be included (see Supplementary 1 for detailed description of clinical assessments). We included *n* = 79 patients in the analysis in Würzburg and *n* = 69 in Münster (for detailed sample description see Supplementary 2).

The study has been conducted in accordance with the Declaration of Helsinki and has been approved by the ethics committees of both participating medical faculties. All participants provided written informed consent and received financial compensation for participation. Furthermore, the study has been pre-registered at ClinicalTrials.gov (registration ID: NCT03208400).

### Study protocol and response assessment

The study protocol is given in Schwarzmeier et al. [49]. Briefly, it comprised five visits (T1: clinical diagnostics and baseline measures, T2: Magnetic Resonance Imaging (MRI), T3: exposure treatment via virtual reality (VRET), T4: clinical post-assessment; T5: 6-month follow-up). VRET comprised psychoeducative information regarding the rationale of exposure treatment and a one-session exposure intervention in virtual reality. Treatment efficacy can be found in Leehr et al. [50]. The primary outcome was defined by a reduction of the sum score of >30% in the German version of the SPQ [48] from pre- to post-treatment. Further, we included a Behavior Avoidance Test (BAT) as a behavioral measure of avoidance as secondary outcome (a reduction of >50% in the initial minimal distance of the patient towards a living bird spider was defined as an indicator for response). The mean within-session fear reduction, resulting from the difference values between maximal and minimal fear in each of the 5 VRET scenarios (0–100) was used as process measure based on prominent learning theories on exposure [51]. Treatment details can be found in Supplementary 3.

### MRI measurements and preprocessing

MR images were acquired using 3-T Siemens Skyra (WZ) and 3-T Siemens Prisma (MS). A T1 structural image was collected via magnetization prepared rapid gradient echo (MPRAGE; matrix = 256 × 256, slices = 176, FOV = 256, voxel size = 1 × 1 × 1 mm, WZ: TE = 2.26 ms, TR = 1.9 s, flip angle = 9°, MS: TE = 2.28 ms, TR 0 2.13 s, flip angle = 8°). During the resting state measurement, lasting 8 min (eyes closed, light switched off), functional images were acquired in ascending order using a T2* weighted echo planar imaging sequence (EPI BOLD; matrix = 64 × 64, slices = 33, FOV = 210, voxel size = 3.3 × 3.3 × 3.8 mm, slice thickness = 3.8 mm, slice gap = 10%, WZ: TE = 30 ms, TR = 2.0 s, flip angle = 90°, MS: TE = 29 ms, TR = 2.0 s, flip angle = 90°). Each slice covered the whole brain and was positioned transaxially parallel to the anterior-posterior commissural line with a tilted angle of 20°.

At each site a harmonized analysis pathway was performed, with different Matlab versions between sites. Structural and functional images were preprocessed in CONN 18a (www.nitrc. org/projects/conn, RRID: SCR_009550) implemented in MATLAB (WZ: R2012b; MS: R2019ba; MathWorks, Natick, Mass) and SPM12 (www.fil.ion.ucl.ac.uk). Due to potential inhomogeneities in initial magnetization the first five functional volumes were discarded. Besides functional realignment and unwarping, preprocessing (CONN default MNI pipeline) included slice-timing correction, structural segmentation (gray matter, white matter, cerebrospinal fluid) and normalization to MNI space, functional normalization to MNI space, and smoothing (5 mm FWHM Gaussian filter). As spurious correlations and thus functional connectivity may be easily introduced by head motions, we used the Artifact Detection Tools (ART, www.nitrc.org/projects/artifact_detect) implemented in CONN to identify outlier images (see Supplementary 2 for the quality control procedure). Patients with less than 10% invalid scans were included and invalid scans were entered as individual 1st-level covariate (scrubbing). For each region of interest (ROI; Automated anatomical labeling atlas, aal, https://www.gin.cnrs.fr/en/tools/aal/, [52]) an average timeseries was extracted from the unsmoothed dataset. Realignment parameters together with their first-order temporal derivatives, patient-specific artefactual covariates (ART-based scrubbing), the effect of rest, and white matter and CSF BOLD time series of each participant were removed from the BOLD signal by applying linear regression to reduce noise. Subsequently, we applied a bandpass-filter to the resulting BOLD time series (bounding box: 0.01Hz- 0.1 Hz) and checked the functional correlations for normal distribution.

### Statistical analyses

On the 1st-level, we performed ROI-to-ROI as well as Seed-to-Voxel analyses of functional connectivity by computing bivariate correlation maps weighted by the hemodynamic response function. Baseline SPQ score, age and duration of exposure were included as 2nd-level covariates of no interest, as there were site and/or group differences. Effects of response group and site were compared using an ANOVA.

For seed-based 2nd-level analyses, we computed the main effect of the respective bilateral seed regions. The cluster-threshold was set to *p* < 0.05 (FDR). The height-threshold was set to *p* < 0.001.

Due to the hypothesis of deficient regulation of the defensive system network via prefrontal structures in non-responders, predefined AAL ROIs were grouped into “defensive system related ROIs” (amygdala, insula, ACC, hippocampus, and thalamus) and “executive control related ROIs” (superior (SFG) and middle frontal gyrus (MFG; including their orbital and medial parts), the IFG pars opercularis, triangularis and orbitalis and the gyrus rectus). For seed-based analyses, we focused on key regions of the defensive system being of utmost relevance in anxiety disorders (the amygdala, the ACC and the hippocampus).

Analyses were performed according to the following three-step protocol: (1) analysis of the presented ROI-to ROI and seed-to-voxel approach on the WZ data (discovery sample) by the WZ team based on three different response criteria, namely SPQ-response (SPQ pre to post reduction> 30%), BAT-response (BAT pre to post reduction> 50%) and low within-session fear reduction (low WS-ext) versus high within-session fear reduction (high WS-ext) using median split); (2) cross-team replication, as it has been shown, that it is by no means trivial to replicate results using the same methods on the same data, but by another team (i.e., analytical reproducibility, [53]): replication of findings from (1) on the WZ data by the MS team; Thus, the aim was to rigorously test our own reporting practices and the effect of different computing environments. (3) cross-site replication: replication from (1) on the MS dataset, to test replicability of results. Additional exploratory analyses using the combined sample (MS and WZ) can be found in Supplementary 7.

## Results

### Sample characteristics

For detailed information regarding the sample characteristics refer to Table 1. The replication sample did not differ from the discovery sample regarding any relevant variable, except for age, age of onset (being correlated with age) and duration of VRET. Besides these differences, clinical characteristics reflected a high homogeneity of the samples. The one session exposure treatment has proved effective in reducing self-reported symptoms and avoidance behavior [50].

**Table 1:** Demographic and clinical characteristics of the included sample at pre-treatment, Means (SD), except where noted. The categorization as responders (vs. non-responders) is based on the primary outcome measure, i.e., SPQ reductions of 30% from pre- to post-treatment assessment.

Variables	Sample Münster			Sample Würzburg			Differences between sites	Difference between response groups at baseline
	All (*n* = 69)	Responder (*n* = 33)	Non-responder (*n* = 36)^a^	All (*n* = 79)	Responder (*n* = 49)^b^	Non-responder (*n* = 30)^c^	Test statistic	Test statistic
Demographic characteristics								
Female gender [*n* (%)]	59 (85.5)	29 (87.9)	30 (83.3)	68 (86.10)	41 (83.7)	27 (90.0)	0.01	0.030
Age (years)	25.83 (6.56)	24.33 (4.85)	27.19 (7.62)	29.16 (9.55)	27.16 (7.47)	32.43 (11.63)	9.561*	9.440*
Years of education	15.22 (2.48)	15.09 (2.35)	15.34 (2.63)	14.35 (3.32)	14.22 (3.37)	14.57 (3.28)	2.545	0.206
Pre-treatment Clinical characteristics								
Age of onset	6.09 (5.08)	5.64 (4.54)	6.50 (5.55)	8.43 (4.06)	9.21 (4.30)	7.14 (3.30)	7.189*	
SPQ	22.59 (1.90)	22.82 (1.81)	22.39 (1.99)	23.17 (2.37)	23.73 (2.32)	22.26 (2.12)	0.757	7.808*
BAT final distance	173.07 (70.64)	166.55 (70.24)	179.06 (71.46)	172.51 (61.55)	181.99 (60.50)	157.02 (61.09)	0.055	0.334
Comorbid major depression [*n* (%)]	1 (1.40)	0 (0.00)	1 (2.80)	1 (1.30)	0 (0.00)	1 (3.30)	2.028	2.550
STAI-Trait	35.10 (7.80)	34.64 (7.87)	35.54 (7.82)	36.85 (9.08)	37.24 (9.07)	36.20 (9.20)	1.613	0.011
BDI-II total	3.18 (3.46)	2.67 (2.62)	3.66 (4.12)	3.68 (4.39)	3.67 (4.38)	3.70 (4.47)	0.855	0.620
Scenarios completed	4.33 (1.20)	4.30 (1.26)	4.36 (1.15)	4.67 (1.01)	4.73 (0.91)	4.57 (1.17)	2.837	0.133
Duration of VRET	76.54 (22.79)	74.94 (25.52)	78.00 (20.24)	87.25 (25.21)	84.98 (24.41)	90.97 (26.46)	7.896*	0.940

Test-statistic = χ2 for categorical and F for metric data; df = 141;

*SPQ* Spider Phobia Questionnaire, *BAT* Behavioral Avoidance Test (smaller values equal smaller distance of the spider to the patient), *STAI-Trait* Trait-version of the State-Trait Anxiety Inventory, *BDI-II* Beck Depression Inventory II.

*p < 0.01.

^a^Years of education, STAI-Trait and BDI-II total reported for *n* = 35;

^b^Age of onset reported for *n* = 48;

^c^Age of onset reported for *n* = 29.

### Analyses on the Würzburg data set (discovery sample)

#### ROI-to-ROI approach

**Fig. 1:**
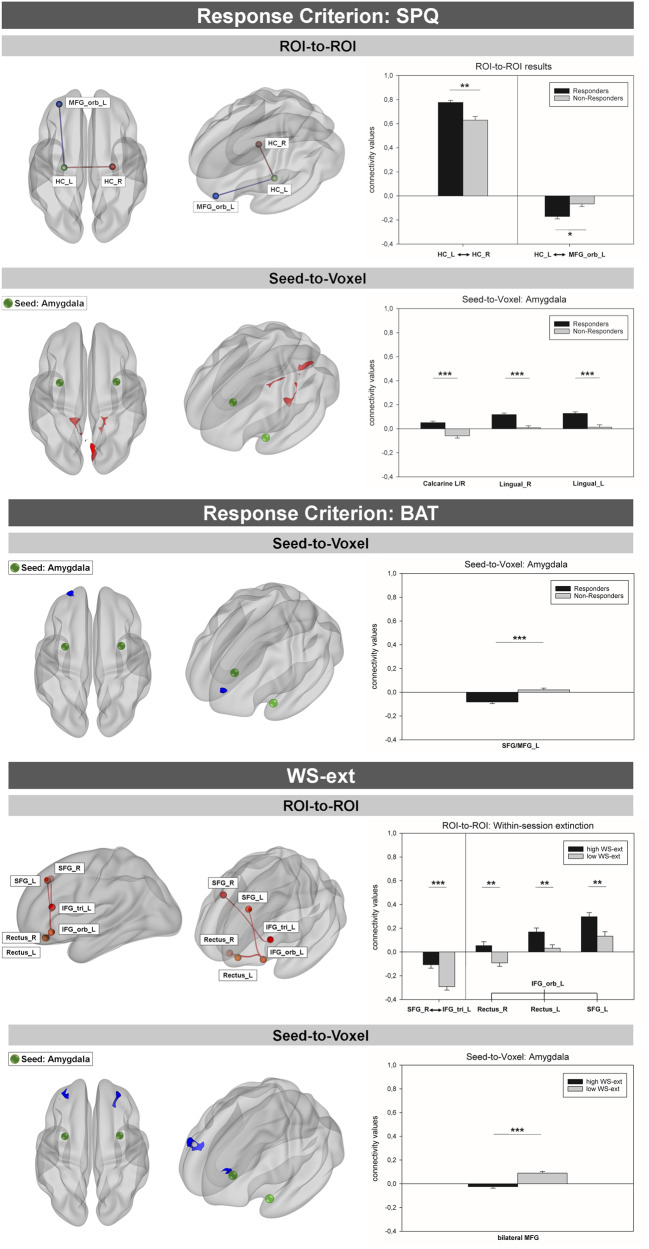
Differential functional connectivity in SPQ- and BAT-responders vs. non-responders and high vs. low WS-ext groups as identified by ROI-to-ROI and Seed-to-Voxel analyses in the Würzburg sample. Clusters/Edges in red indicate responders to exhibit stronger positive connectivity compared to non-responders. Clusters/Edges in blue indicate stronger negative connectivity in responders. The corresponding bar graphs show connectivity values (Pearson’s correlation coefficients extracted from the respective cluster(s)/edges. *ROI-to-ROI:* Spheres indicate ROIs. Edges indicate significant connectivity between ROIs. *Seed-to-Voxel:* Green spheres indicate seeds. SPQ Spider Phobia Questionnaire, BAT Behavior Avoidance Test, WS-ext within-session fear reduction, L left, R right, ROI Region of Interest, MFG_orb middle frontal gyrus pars orbitalis, HC Hippocampus, SFG superior frontal gyrus, MFG middle frontal gyrus, cluster threshold: *p* < 0.05 (FDR); height-threshold: *p* < 0.001 (uncorr.); *p < 0.05; **p < 0.01; ***p < 0.001.

The comparison of SPQ-responders to SPQ-Non-responders revealed a differential connection of two brain regions to the left hippocampus: There was significantly increased negative functional connectivity between the orbital part of the left MFG and left hippocampus in responders, T(74) = −3.38, *p* < 0.05, *d* = −0.78. Furthermore, left and right hippocampus exhibited increased positive connectivity among responders, T(74) = 3.77, *p* < 0.01, *d* = 0.87 (see Fig. 1 and Table 2).

**Table 2:** Differential functional resting-state connectivity within SPQ-responders and non-responders (ROI-to-ROI and Seed-to-Voxel).

Analyses discovery sample								Cross-team replication									Cross-site replication								
ROI-to-ROI						T	p_FDR_								T	p_FDR_								T	p_FDR_
*t-contrast: resp > non-resp*																									
Hippocampus L ⟷		Hippocampus R				3.77	<0.01	Hippocampus L ⟷			Hippocampus R				3.83	<0.01	Middle frontal gyrus, medial part R ⟷			Middle frontal gyrus, orbital part R				3.53	<0.05
								Inferior frontal gyrus, pars opercularis L ⟷			Inferior frontal gyrus, pars triangularis R				3.32	<0.05	Insula L ⟷			Thalamus R				− 3.72	<0.05
*t-contrast: non-resp > resp*																									
Hippocampus L ⟷		Inferior frontal gyrus, pars orbitalis				3.38	<0.05	Hippocampus L ⟷			Inferior frontal gyrus, pars orbitalis				3.65	<0.05									
Seed-to-Voxel	Side	k	x	y	z	T	p_FDR_			Side	k	x	y	z	T	p_FDR_		Side	x	y	z			T	p_FDR_
Amygdala																									
*t-contrast: resp > non-resp*																									
*Cluster 1:*		174	6	−88	6	4.85	<0.001	*Cluster 1:*			137	6	−86	6	3.20	<0.05	No differential connectivity								
Calcarine	R	99						Calcarine		R	88														
Lingual gyrus	R	46						Lingual gyrus		R	35														
	L	17						Calcarine		L	12														
Calcarine	L	11						Lingual gyrus		L	2														
AAL not-labeled	--	1																							
*Cluster 2*:		151	−10	−42	−12	5.27	<0.001																		
Cerebellum 4/5	L	105																							
Lingual gyrus	L	25																							
Fusiform gyrus	L	14																							
Vermis 4/5	--	5																							
Cerebellum 3	L	2																							
*Cluster 3:*		108	16	−52	−10	4.93	<0.001																		
Cerebellum 4/5	R	58																							
Fusiform gyrus	R	24																							
Lingual gyrus	R	17																							
Cerebellum 6	R	9																							
Seed-to-Voxel	Side	k	x	y	z	T	p_FDR_			Side	k	x	y	z	T	p_FDR_		Side	x	y	z			T	p_FDR_
*t-contrast: non-resp > resp*																									
No differential connectivity																	No differential connectivity								
Seed-to-Voxel	Side	k	x	y	z	T	p_FDR_			Side	k	x	y	z	T	p_FDR_		Side	x	y	z			T	p_FDR_
Anterior cingulate cortex																									
*t-contrast: resp > non-resp*																									
*Cluster 1:*		158	−14	28	10	6.32	<0.001	*Cluster 1:*			175	−12	28	8	3.2	<0.001	No differential connectivity								
Anterior cingulate cortex	L							Anterior cingulate cortex		L	24														
	R									R	6														
Mid cingulate cortex	L							Mid cingulate cortex		L	3														
	R									R	3														
AAL not-labeled	--							AAL not-labeled		--	139														
*t-contrast: non-resp > resp*																									
No differential connectivity								No differential connectivity									No differential connectivity								
Hippocampus																									
*t-contrast: resp > non-resp*																									
Cluster 1:		102	26	−78	−14	4.00	<0.001	*Cluster 1:*			118	26	−78	−14	3.2	<0.05	No differential connectivity								
Lingual gyrus	R	72						Lingual gyrus			95														
Fusiform gyrus	R	25						Fusiform gyrus			18														
Cerebellum 6	R	5						Cerebelum 6			5														
*t-contrast: non-resp > resp*																									
No differential connectivity								No differential connectivity									No differential connectivity								

*FDR* false discovery rate, *k* number of voxels per cluster/region, *non-resp* non-responder, *resp* responder, *ROI* region of interest, *SPQ* spider phobia questionnaire, x, y, z, MNI coordinates.

We observed no differential functional connectivity between BAT-responders and BAT-non-responders with respect to ROI-to-ROI analyses (see Supplementary 4 Table S1).

When comparing patients with high vs. low WS-ext values we found significantly decreased negative connectivity between the right SFG and left triangular IFG in patients exhibiting more fear reduction during exposure treatment, T(74) = 4.36, *p* < 0.001, *d* = 1.01. Additionally, positive connectivity between the left orbital IFG and the bilateral gyrus rectus, left: T(74) = 3.02, *p* < 0.01, *d* = 0.70, right: T(74) = 3.23, *p* < 0.01, *d* = 0.75, and the left SFG was significantly increased in patients showing more fear reduction during exposure, T(74) = 3.17, *p* < 0.01, *d* = 0.74 (see Supplementary 5 Table S2).

#### Seed-to-voxel approach

##### Amygdala

The bilateral amygdalae were significantly higher positively connected to three adjacent clusters within the occipital cortex and the occipito-temporal junction in SPQ-responders. The first cluster was located within the medial occipital cortex and comprised the bilateral calcarine and lingual gyri, T(74) = 4.85, *p* < 0.001, *d* = 1.12. The two further clusters were lateralized but mirrored each other congruently. They extended over the lingual gyrus, cerebellar lobules 4 and 5 and parts of the fusiform gyrus, right: T(74) = 5.27, *p* < 0.001, *d* = 1.22; left: T(74) = 4.93, *p* < 0.001, *d* = 1.14 (see Table 2 and Fig. 1).

When using the BAT as response criterion, we found activity in a cluster extending over the left superior and middle frontal gyrus to be stronger negatively associated with amygdala activity in responders compared to non-responders, T(74) = −4.91, *p* < 0.001, *d* = −1.14 (see Supplementary 4 Table S1 and Fig. 1).

Seed-to-voxel analyses of the amygdala revealed the bilateral amygdalae to be connected significantly less positive with the bilateral MFG and the left SFG in patients exhibiting more anxiety reduction during exposure, T(74) = −5.57, *p* < 0.001, *d* = −1.29 (see Supplementary 5 Table S2).

##### Anterior cingulate cortex

We observed significantly increased positive connectivity between bilateral ACC seeds among SPQ-responders. The cluster predominantly comprised the left anterior and mid cingulum but also portions of the corresponding regions within the right hemisphere, T(74) = 6.32, *p* < 0.001, *d* = 1.47 (see Table 2).

No results were found using the secondary outcome or within-session fear reduction as response criteria (see Supplementary 4 Table S1 and Supplementary 5 Table S2).

##### Hippocampus

Among responders, the bilateral hippocampi exhibited significantly increased positive connectivity with a cluster at the junction of occipital and temporal lobe. It comprised parts of the right lingual and fusiform gyrus but also of the sixth cerebellar lobule, T(74) = 4.00, *p* < 0.001, *d* = 0.93 (see Table 2).

No results were found using the secondary outcome or within-session fear reduction as response criterion (see Supplementary 4 Table S1 and Supplementary 5 Table S2).

### Cross-team replication on the Würzburg data set

#### ROI-to-ROI approach

Cross-team replication of the ROI-to-ROI results supported the increased positive connectivity between the bilateral hippocampi in SPQ-responders, compared to non-responders, T(74) = 3.83, *p* < 0.01, *d* = 0.89, and decreased negative functional connectivity between the left hippocampus and the orbital part of the left MFG, T(74) = −3.32, *p* < 0.05, *d* = −0.77. In contrast to the analyses in Würzburg, cross-team analyses further showed an increased positive connectivity in responders between the left IFG pars opercularis and right IFG pars triangularis, T(74) = 3.65, *p* < 0.05, *d* = 0.85.

As in the discovery sample, no differential functional connectivity between BAT-responders and BAT-non-responders with respect to ROI-to-ROI analyses could be revealed (see Supplementary 4 Table S1).

Cross-team replication of the ROI-to-ROI results comparing patients with high vs. low WS-ext succeeded (see Supplementary 5 Table S2). Thus, we were able to replicate the findings of decreased negative connectivity between SFG and triangular IFG, and increased positive connectivity between the left orbital IFG and the bilateral rectus in patients with more within-session fear reduction during exposure treatment.

#### Seed-to-Voxel approach

##### Amygdala

Cross team replication only yielded one cluster comprising the intracalcarine cortex, the lingual gyrus, as well as the occipital pole, significantly higher positively connected to the bilateral amygdalae: T(74) = 3.20, *p* = 0.0113, *d* = 0.74 (see Table 2). The two other clusters could be replicated by slightly lowering the cluster threshold to *p* < 0.06 (compare Supplementary 6 Table S3).

We replicated the decreased negative association in BAT-responders compared to non-responder between the amygdala and a cluster extending over the left superior and middle frontal gyrus, T(74) = −3.2, *p* < 0.05, *d* = −0.74 (see Supplementary 4 Table S1).

Cross-team replication revealed only one cluster being less positively connected in patients exhibiting high WS-ext compared to the low WS-ext group, T(74) = −3.2, *p* < 0.01, *d* = −0.74 (see Supplementary 5 Table S2).

##### Anterior cingulate cortex

Corroborating results from the Würzburg team, responders exhibited significantly increased positive connectivity between bilateral ACC seeds resulting in a cluster predominantly comprising the left anterior and mid cingulum but also small parts of the right anterior and mid cingulum, T(74) = 3.20, *p* < 0.001, *d* = 0.74 (see Table 2).

As in the primary analyses, no results were found using the secondary outcome or within-session fear reduction as response criteria (see Supplementary 4 Table S1 and Supplementary 5 Table S2).

##### Hippocampus

The bilateral hippocampi showed significantly increased positive connectivity with one cluster localized at the border of occipital and temporal lobe. It extended over the right lingual and fusiform gyrus but also small parts of the sixth cerebellar lobule, T(74) = 4.00, *p* < 0.001, *d* = 0.93.

No results were found using the secondary outcome or within-session fear reduction as response criteria (see Supplementary 4 Table S1 and Supplementary 5 Table S2).

### Cross-site replication on the Münster data set

#### ROI-to-ROI approach

Instead of increased negative functional connectivity between the orbital part of the left MFG and left hippocampus and the increased positive functional connectivity within the hippocampus in SPQ-responders from the discovery sample, we observed increased positive connectivity in SPQ-responders between the medial and the orbital part of the right MFG, T(64) = 3.53, *p* < 0.05, *d* = 0.85 and between the left insula and the right thalamus, T(64) = 3.72, *p* < 0.05, *d* = 0.90, compared to SPQ-non-responders (see Table 2).

Neither the BAT nor the within-session fear reduction response criterion revealed any results in the replication sample (see Supplementary 4 Table S1 and Supplementary 5 Table S2).

#### Seed-to-Voxel approach

##### Amygdala

Cross-site replication did not show any differential connectivity based on an amygdala seed approach. Neither the BAT nor the within-session fear reduction response criterion revealed any results in the replication sample.

##### Anterior cingulate cortex

Seed-based analyses with the ACC as seed region revealed no differential connectivity in the replication sample.

BAT-responders compared to non-responders showed increased positive connectivity between ACC and left thalamus, T(64) = 3.22, *p* < 0.001, *d* = 0.78. BAT-non-responders showed increased positive connectivity between the ACC and a cluster including a small part of the caudate nucleus, T(64) = 3.22, *p* < 0.05, *d* = 0.78.

No differential connectivity was found regarding the within-session fear reduction criterion.

##### Hippocampus

No connectivity differences between responders and non-responders could be found for the Hippocampus.

BAT-responders did not show differential connectivity compared to BAT-non-responders, except for a small cluster (k = 2) indicating that BAT-responders show increased positive connectivity between the hippocampus and the Cerebellum, T(64) = 3.22, *p* < 0.05, *d* = 0.78.

Patients with high WS-ext showed increased positive connectivity between the hippocampus seed and an occipital cluster comprising parts of the superior occipital cortex, the cuneus, T(64) = 3.22, *p* < 0.05, *d* = 0.78 and a second cluster comprising the right lingual and the calcarine gyrus, T(64) = 3.22, *p* < 0.05, *d* = 0.78.

## Discussion

The present investigation aimed to identify pre-treatment resting-state connectivity signatures that moderate response towards exposure as a first-line treatment for specific phobia. Analyses were embedded within a strict replication approach. Main findings on moderators of treatment response in the discovery sample suggest: (a) increased inhibitory fronto-limbic rsFC, (b) increased positive amygdala-visual rsFC, and (c) more excitatory “cross-talk” between prefrontal regions in patients with a high within-session fear extinction. Regarding the replication, results were mixed: while the cross-team replication showed largely comparable results, replicating findings in the replication sample was not successful, highlighting the importance of further replications.

In line with our overarching hypothesis, SPQ-responders in the discovery sample exhibited increased inhibitory fronto-limbic connectivity between left hippocampus and left orbital MFG and increased positive connectivity between the bilateral hippocampi. Those results could be interpreted with respect to hippocampal replay, which has been related to rumination and worry in anxiety disorders and depression [54], memory-guided behavior [55] as well as the retrieval of (fear) memory contents in rodents and humans [56–59]. It is defined as “the rapid, coordinated reactivation of encoding-activated cellular ensembles during sleep and resting wakefulness” [54]. In rodents, an inhibition of ventral hippocampal projections to the medial PFC via an optogenetic approach seems to disrupt anxiety and avoidance behavior [60]. In contrast, increased synchrony between hippocampus and medial PFC has been observed during states of anxiety [61]. The present results might therefore indicate altered retrieval of fear-relevant memory contents among patients who will benefit well from exposure. This may support the acquisition of a new fear-inhibitory memory trace or facilitate competing with the old fear-related memory trace thus resulting in better treatment success.

Findings on stronger fronto-limbic connectivity as a prerequisite for treatment response were also reflected in our secondary, more behavioral outcome measure (BAT response), where frontal activity in the left superior and middle frontal gyrus was more negatively associated with amygdala activity in responders compared to non-responders.

Supplementing our primary hypothesis on fronto-limbic connectivity, treatment responders in the discovery sample were additionally characterized by increased positive limbic-occipital to temporal connectivity: the amygdala and hippocampus exhibited significantly increased positive connectivity with clusters in the occipital and posterior temporal lobe in responders following the bilateral ventral visual pathway [62, 63]. Previous findings on backward projections of the amygdala to visual cortices suggest this connection to account for saliency of emotional visual information [64–69]. Within this framework, responders seem to be characterized by pronounced amygdala-driven processing of fear-relevant stimuli within the visual cortex, which may facilitate information processing within fear-relevant structures and stronger inhibitory fronto-limbic connectivity. Moreover, this may lead to attenuated fear memory retrieval. This interpretation is supported by recent findings on effective connectivity between amygdala, hippocampus and VMPFC and their interplay with respect to the elaboration and retrieval of autobiographical memories [70]. Here, hippocampus-VMPFC connectivity was increased when reliving emotionally arousing events.

Additionally, we exploratively introduced within-session fear reduction as a process measure of inhibitory learning. We found dorsolateral and ventromedial prefrontal areas to be stronger connected to the orbital IFG in high WS-ext patients compared to low WS-ext patients. Additionally, the right SFG was less inhibitorily connected to the triangular IFG in high WS-ext patients. Repeatedly, dorsolateral, ventrolateral and ventromedial prefrontal areas have been named as neural substrates of fear extinction and anxiety disorders [71–74]. The structures further correspond to the three steps of (voluntary) emotion regulation as suggested by the extended process model of emotion regulation [75]: the evaluation of the need to regulate (VLPFC [75]); the selection of adequate regulation strategies plus their implementation (DLPFC [76, 77]); Heightened connectivity among those regions might therefore reflect enhanced reconciliation and balancing of those processes [78], which may facilitate extinction during subsequent exposure [79, 80].

When comparing high and low WS-ext groups with respect to seed-to-voxel connectivity of the amygdala significant differential connectivity with the bilateral MFG was identified as it was true for the same analysis among BAT responders and non-responders. Profound investigation of the absolute connectivity values suggests that increased connectivity between amygdala and MFG within the low WS-ext group may indicate a heightened need for regulatory control.

As expected, cross-team replication, produced largely overlapping results. Of note, some results could only be replicated by lowering the cluster threshold to *p* < 0.06, thus the performance of the same analyses still seems to add some variance to the results. This is a common finding in reproducibility research [81] and can be traced back to the lack of standardized reporting practices, sharing analysing codes, and differences in the computing environment. In our case, we did not use the same code, but only written reports to transfer analysing methods and used different computing environments.

In line with the so-called replication crisis, the results from the discovery sample could not be replicated in the independent replication sample. This warrants an explanation. Firstly, only few cross-site replications of rsCF results in anxiety disorders exist [82], thus the generalizability of the results from one sample to another remains unclear. Besides, replication literature might be subject to a strong publication bias, hence lacking publication of unsuccessful replication studies. Further, studies are needed that dive deeper into the sources of variance that might affect replication. In our case, different scanner types faced a highly harmonized protocol regarding assessment and pre-processing of resting-state data. A potential reason for our replication failure may be the rather small sample sizes [83], which are however quite large for clinical neuroimaging studies (for results using the combined sample see Supplementary 7). Marek et al.[83]. suggest reproducibility of brain-wide association studies to require samples with thousands of individuals, while further stating that reproducibility also depends on the effect sizes. Regarding our discovery sample, we had large effects ranging from Cohen’s d 0.07 to 1.47, which resulted in a theoretical minimal power of 89% in a sample of the size of the replication sample. As sample sizes as proposed by Marek et al. [83] may not be feasible within single clinical studies, there is a high need for research initiatives to develop harmonized analysis protocols [84, 85] and to pool data to achieve sufficient statistical power [83], e.g., as promoted by the ENIGMA consortium (https://enigma.ini.usc.edu/).

All this opens the field to a broader discussion regarding the means to increase transparency and reproducibility in research, which has gained upwind the last couple of years, e. g. registered reports [86] and open science [87].

Taken the inconclusive interpretation of our cross-site replication approach, deviant results in the replication sample also need to be discussed. We found limbic and frontal regions to be stronger interconnected in responders. Especially the increased positive connectivity between the thalamus and the insula might be of interest. The insula is regarded as integration hub for emotional and interoceptive information [88], whereas the thalamus is the main input of bottom-up stimuli information before being transferred to the cortex and is involved in prefrontal inhibition processes relevant in anxiety [89]. Further, both regions belong to the extended defensive system [90]. Corroborating the finding of stronger interconnection of limbic structures in SPQ-responders, BAT-responders also showed increased limbic interconnection. BAT-non-response was associated with increased positive connectivity of the defensive system with the caudate nucleus (Supplementary Table S1). In line with the primary results of increased connectivity of limbic structures with the visual cortices in responders, high within-session fear reduction was associated with increased connectivity between limbic structures and the visual system (Supplementary Table S2).

Interpretation of the results requires the consideration of limitations. Due to strict inclusion/exclusion criteria, the present investigation was based on a rather homogeneous patient sample, resulting in high internal validity but limiting generalizability to clinical samples. We did control for variables showing differences between the two samples. Generalizability is further limited by the use of VRET, which allowed for high experimental control and standardization in the execution of exposure as well as the application of the very same exposure for each participant on the one hand, but involved characteristics (e.g., disconfirmation of certain beliefs is not possible in VR) that might have condensed within treatment response and the associated pre-treatment characteristics and restricted individualization of exposure on the other. However, as VRET relies on the same mechanism of action as in-vivo exposure and has been demonstrated to be equally effective [91], we believe the use of VRET does not substantially impair the transfer of our findings toward traditional exposure-based CBT.

The comparison of the observed results of the two response criteria as well as regarding WS-ext shows an overlap with respect to inhibitory fronto-limbic connectivity, which characterizes responders and high WS-ext patients. However, results also differ substantially between classification methods. As SPQ and BAT groups overlap for only about 61.22%, and overlap is even smaller with the WS-ext groups differences in group composition might have led to differing results. Future studies should try to achieve a more comprehensive understanding of the individual aspects covered by the different criteria for classifying treatment response [1].

Furthermore, replication is necessary, also with respect to treatments beyond a one-session exposure as well as within other anxiety disorders.

As cross-site replication of our findings within a second independent sample was not successful even though the prerequisite of a prior successful cross-team replication was met and study setup and data analysis pathways were fully harmonized. On the other hand, published replication data are rarely available, thus limiting the interpretation of findings in terms of replication results in general. Clearly, more studies are needed that explicitly address the replicability of their findings.

Clinically, findings in the discovery sample suggest that treatment adaptations supporting inhibitory fronto-limbic connectivity might enhance response rates. This might be achieved e.g., via the implementation of an emotion regulation training prior to treatment (see e.g., [92, 93]), frontal neurofeedback [94–96], or the application of gamma amino butyric acid (GABA) related drugs [97, 98]. Furthermore, brain stimulation techniques like repetitive transcranial magnetic stimulation (rTMS) as well as transcranial direct current stimulation (tDCS) may constitute options for directly manipulating brain activity and thus also connectivity (see e.g., [99–103]). First evidence suggests an effect of tDCS stimulation of the ventromedial prefrontal cortex on neural fear generalization patterns in a non-clinical sample [104]. Still, as we were not able to replicate results, interpretation remains inconclusive.

In summary, we identified three potential pre-treatment signatures of treatment response in large-scale intrinsic brain networks: i) inhibitory fronto-limbic connectivity which may confer emotion regulation via fear-inhibitory learning, ii) pronounced visuo-limbic connectivity which may be a clinical correlate of reduced avoidance behavior, and iii) enhanced crosstalk within the dorsolateral to ventromedial PFC supporting fear reduction during the exposure process, possibly via conscious and/or verbal cognitive processing of contingencies. Those three patient features may represent three distinct routes to facilitate fear reduction via exposure and hence inform treatment augmentation strategies for patients particularly vulnerable for treatment non-response. Findings are however limited by the lack of cross-site replication and warrant critical reflection as well as considerations how to foster replications in clinical neuroimaging studies.

### Supplementary information

Supplementary Material

## Data Availability

The study protocol of the study is published (Schwarzmeier et al., [49]). The data are not publicly available due to privacy or ethical restrictions.
