# Supplementary material for: Association between resting-state connectivity patterns in the defensive system network and treatment response in spider phobia—a replication approach
Source: Transl Psychiatry. 2024 Mar 7;14:137. doi: 10.1038/s41398-024-02799-x (PMC10920691; doi:10.1038/s41398-024-02799-x)
Supplement: Supplementary file 1 — Supplementary Material [file 41398_2024_2799_MOESM1_ESM.docx]

**Supplement: „Association between resting-state connectivity patterns in the defensive system and treatment response in spider phobia – a cross-team and cross-sample replication approach”**

Supplement 1: Clinical assessments

A German translation of the Spider Phobia Questionnaire (Klorman et al., 1974) was determined to be the primary outcome measure. This questionnaire is recommended for diagnostics of spider phobia within the treatment manual of Hamm (2006) and has been shown to exhibit satisfactory quality criteria e.g. with respect to test-retest-reliability (Muris & Merckelbach, 1996). A SPQ-score of 20 and higher, which previously has been proposed as cut-off to indicate clinical significance of symptom severity (Hamm, 2006; Öst, 1996), was mandatory for inclusion. Further, patients had to be right-handed, of Caucasian descent, without a lifetime diagnosis of comorbid mental disorders except for mild to moderate depression (unless currently treated psychotherapeutically or pharmacologically) and further specific phobias of the animal subtype. A current pregnancy or fulfilment of MRI-contraindications led to exclusion. To be classified as a responder, a patient had to exhibit at least 30% SPQ score reduction from baseline to post assessment. An in vivo Behavioral Avoidance Test (BAT) served to determine the behavioral component of response, which served as secondary outcome measure. A plastic box with a living bird spider (*Grammostola rosea*) was put on a wooden slide. Participants had to drag the box towards themselves as close as possible by using a crank. The remaining distance between patient and the box served to quantify avoidance behavior (in cm). BAT-treatment responders had to exhibit a distance reduction of at least 50% from baseline to post assessment or follow-up, respectively.

Within-session fear reduction was assessed as mean of the difference values of maximal and minimal self-reported fear in each scenario of the virtual reality exposure treatment.

Supplement 2: sample composition and quality control procedure

The sample used in this study is a sub-sample from the study Leehr et al. (2021). From the N= 87 participant in Würzburg, with data available at pre and post assessment, n= 2 had to be excluded from our resting-state analyses due to brain structural abnormalities and n= 6 due to an invalid scan ratio> 10%. In Münster, data of pre- and post-assessment was available for N= 87 patients, but n= 18 had to be excluded from the resting-state-analyses due to an invalid scan ratio> 10%. We considered an image an invalid scan (conservative settings, 95th percentile) if frame wise head displacement exceeded 0.5 mm in any direction (x,y,z), or if global mean intensity (GSI) for the respective image was more than three standard deviations from mean GSI for the entire resting-state scan. A ratio of more than 10% of invalid scans (here: > 23) within one patient led to exclusion from further analyses. Following preprocessing, we visually checked normalization of structural and functional images to MNI space for each patient via CONN’s “QA_NORM” function. Additionally, structural segmentation was separately inspected for each patient via an overlay with the mask outline.

Supplement 3: Virtual reality exposure treatment (VRET)

The virtual environment was rendered via the research systems software of VT+ (VTplus GmbH, Würzburg, Germany) and subsequently generated by the Steam Source engine (Valve Corp., Bellevue, Washington, USA). The VR scenarios were displayed via a Z800 3D Visor head-mounted display (HMD; eMagin, NY, USA) in Würzburg and an Oculus Rift DK2 (HMD; Oculus VR, LLC) in Münster. Each patient underwent the same five scenarios in a manualized manner with a maximal overall duration of 2.5 hours. To support generalization, the scenarios differed with respect to size, number, positioning and movement of the virtual spiders. For a detailed description of the different scenarios please refer to Schwarzmeier et al. (2019). Participants were asked to approach the spider(s) in each scenario and refrain from avoidance behavior. A scenario was seen as completed if the subjective units of distress (SUD) dropped below 20 or stagnated at least three times consecutively on a higher level.

Supplement 4

***Table S1.*** Differential functional resting-state connectivity within BAT-responders and non-responders (ROI-to-ROI and Seed-to-Voxel).

| **Primary analyses** | | | | |  |  |  |  |  |  | **Cross-team replication** | | | |  |  |  |  |  |  | **Cross-site replication** | | | |  |  |  |  |  |
| --- | --- | --- | --- | --- | --- | --- | --- | --- | --- | --- | --- | --- | --- | --- | --- | --- | --- | --- | --- | --- | --- | --- | --- | --- | --- | --- | --- | --- | --- |
|  | | | | |  |  |  |  |  |  |  | | | |  |  |  |  |  |  |  | | | |  |  |  |  |  |
| **ROI-to-ROI** | |  |  | |  |  |  | T | p_FDR_ | |  |  |  |  |  |  |  | T | p_FDR_ | |  |  |  |  |  |  |  | T | p_FDR_ |
|  | |  |  | |  |  |  |  |  | |  |  |  |  |  |  |  |  |  | |  |  |  |  |  |  |  |  |  |
| *t-contrast: resp > non-resp* | | | | |  |  |  |  |  |  |  |  |  |  |  |  |  |  |  |  |  |  |  |  |  |  |  |  |  |
|  | | | | |  |  |  |  |  |  |  |  |  |  |  |  |  |  |  |  |  |  |  |  |  |  |  |  |  |
| No differential connectivity | | | | | | |  |  |  | | No differential connectivity | | | | | |  |  |  | | No differential connectivity | | | | | |  |  |  |
|  | | |  | | | |  |  |  | |  | | |  | | |  |  |  | |  | | |  | | |  |  |  |
| *t-contrast: non-resp > resp* | | | | |  |  |  |  |  |  |  |  |  |  |  |  |  |  |  |  |  |  |  |  |  |  |  |  |  |
|  | | | | |  |  |  |  |  |  |  |  |  |  |  |  |  |  |  |  |  |  |  |  |  |  |  |  |  |
| No differential connectivity | | | | | | |  |  |  | | No differential connectivity | | | | | |  |  |  | | No differential connectivity | | | |  |  |  |  |  |
|  | |  |  | |  |  |  |  |  | |  |  |  |  |  |  |  |  |  | |  |  |  |  |  |  |  |  |  |
| **Seed-to-Voxel** | | Side | k | | x | y | z | T | p_FDR_ | |  |  | Side | k | x | y | z | T | p_FDR_ | |  |  | Side | k | x | y | z | T | p_FDR_ |
|  | |  |  | |  |  |  |  |  |  |  | |  |  |  |  |  |  |  |  |  |  |  |  |  |  |  |  |  |
| Amygdala | |  |  | |  |  |  |  |  |  |  | |  |  |  |  |  |  |  |  |  |  |  |  |  |  |  |  |  |
| *t-contrast: resp > non-resp* | | | | |  |  |  |  |  |  |  | | | |  |  |  |  |  |  |  |  |  |  |  |  |  |  |  |
|  | | | | |  |  |  |  |  |  |  | | | |  |  |  |  |  |  |  |  |  |  |  |  |  |  |  |
| No differential connectivity | | | | |  |  |  |  |  | | No differential connectivity | | | |  |  |  |  |  | |  | | | |  |  |  |  |  |
|  | |  |  | |  |  |  |  |  |  |  | |  |  |  |  |  |  |  |  |  |  |  |  |  |  |  |  |  |
| *t-contrast: non-resp > resp* | | | | | |  |  |  |  |  |  |  |  |  |  |  |  |  |  |  |  |  |  |  |  |  |  |  |  |
| *Cluster 1* |  | | | 88 | -26 | 60 | 20 | 4.91 | <.001 | | *Cluster 1* | |  | 91 | -26 | 60 | 20 | 3.2 | <.05 | | No differential connectivity | | | |  |  |  |  |  |
| Superior frontal gyrus | L | | | 67 |  |  |  |  |  |  | Superior frontal gyrus | | L | 70 |  |  |  |  |  | |  | | | |  |  |  |  |  |
| Middle frontal gyrus | L | | | 21 |  |  |  |  |  |  | Middle frontal gyrus | | L | 21 |  |  |  |  |  |  |  | | | |  |  |  |  |  |
|  | | | | |  |  |  |  |  |  |  |  |  |  |  |  |  |  |  |  |  |  |  |  |  |  |  |  |  |
| Anterior cingulate cortex | | | | |  |  |  |  |  |  |  |  |  |  |  |  |  |  |  |  |  |  |  |  |  |  |  |  |  |
| *t-contrast: resp > non-resp* | | | | | |  |  |  |  |  |  |  |  |  |  |  |  |  |  |  |  |  |  |  |  |  |  |  |  |
|  | | | | | |  |  |  |  |  |  |  |  |  |  |  |  |  |  |  |  |  |  |  |  |  |  |  |  |
| No differential connectivity | | | | |  |  |  |  |  | |  | |  |  |  |  |  |  |  | | *Cluster 1* | |  | 205 | -6 | -16 | 6 | 3.22 | <.001 |
|  | | | | |  |  |  |  |  | |  | |  |  |  |  |  |  |  | | Thalamus | | L | 156 |  |  |  |  |  |
|  | | | | |  |  |  |  |  | |  | |  |  |  |  |  |  |  | | unlabeled | | -- | 49 |  |  |  |  |  |
|  | | | | |  |  |  |  |  | |  | |  |  |  |  |  |  |  | |  | | | |  |  |  |  |  |
| *t-contrast: non-resp > resp* | | | | | |  |  |  |  |  |  |  |  |  |  |  |  |  |  |  |  |  |  |  |  |  |  |  |  |
|  | | | | | |  |  |  |  |  |  |  |  |  |  |  |  |  |  |  |  |  |  |  |  |  |  |  |  |
| No differential connectivity | | | | |  |  |  |  |  |  |  | | | |  |  |  |  |  |  | *Cluster 1* | |  | 120 | 22 | 28 | 0 | 3.22 | <.05 |
|  | | | | |  |  |  |  |  |  |  | | | |  |  |  |  |  |  | Nucleus caudatus | | R | 14 |  |  |  |  |  |
|  | | | | |  |  |  |  |  |  |  | | | |  |  |  |  |  |  | Putamen | | R | 2 |  |  |  |  |  |
|  | | | | |  |  |  |  |  |  |  | | | |  |  |  |  |  |  | Frontal Superior Cortex | | R | 1 |  |  |  |  |  |
|  | | | | |  |  |  |  |  |  |  | | | |  |  |  |  |  |  | Unlabeled | | -- | 103 |  |  |  |  |  |

| *Table S1 to be continued.* | | | | |  |  |  |  |  | | | |  |  |  |  |  |  |  |  |  |  |  |  |  |  |  |
| --- | --- | --- | --- | --- | --- | --- | --- | --- | --- | --- | --- | --- | --- | --- | --- | --- | --- | --- | --- | --- | --- | --- | --- | --- | --- | --- | --- |
| **Primary analyses** | | |  |  |  |  |  |  | **Cross-team replication** | | | |  |  |  |  |  |  | **Cross-site replication** | | | |  |  |  |  |  |
|  | | |  |  |  |  |  |  |  | | | |  |  |  |  |  |  |  | | | |  |  |  |  |  |
| **Seed-to-Voxel** | Side | k | x | y | z | T | p_FDR_ | |  |  | Side | k | x | y | z | T | p_FDR_ | |  |  | Side | k | x | y | z | T | p_FDR_ |
| Hippocampus | |  |  |  |  |  |  |  |  |  |  |  |  |  |  |  |  |  |  |  |  |  |  |  |  |  |  |
| *t-contrast: resp > non-resp* | | |  |  |  |  |  |  |  |  |  |  |  |  |  |  |  |  |  |  |  |  |  |  |  |  |  |
|  | | |  |  |  |  |  |  |  |  |  |  |  |  |  |  |  |  |  |  |  |  |  |  |  |  |  |
| No differential connectivity | | |  |  |  |  |  | |  | |  |  |  |  |  |  |  | | *Cluster 1* | |  | 87 | 16 | -42 | -36 | 3.22 | <.05 |
|  |  |  |  |  |  |  |  | |  | |  |  |  |  |  |  |  |  | Cerebellum 9 | |  | 2 |  |  |  |  |  |
|  |  |  |  |  |  |  |  | |  | |  |  |  |  |  |  |  |  | Unlabeled | |  | 95 |  |  |  |  |  |
|  |  |  |  |  |  |  |  | |  | |  |  |  |  |  |  |  |  |  |  |  |  |  |  |  |  |  |
| *t-contrast: non-resp > resp* | | |  |  |  |  |  |  |  |  |  |  |  |  |  |  |  |  |  |  |  |  |  |  |  |  |  |
|  | | |  |  |  |  |  |  |  |  |  |  |  |  |  |  |  |  |  |  |  |  |  |  |  |  |  |
| No differential connectivity | | |  |  |  |  |  |  |  | | | |  |  |  |  |  |  | No differential connectivity | | | |  |  |  |  |  |

*Notes:* FDR= False Discovery Rate; k= number of voxels per cluster/region; non-resp= non-responder; resp= responder; ROI= Region of Interest, SPQ= Spider Phobia Questionnaire; x, y, z, MNI coordinates.

Supplement 5

***Table S2.*** Differential functional resting-state connectivity within high versus low within session fear reduction (ROI-to-ROI and Seed-to-Voxel).

| **Primary analyses** | | | | | |  |  |  |  |  |  | **Cross-team replication** | | | |  |  |  |  |  |  | **Cross-site replication** | | | |  |  |  |  |  |
| --- | --- | --- | --- | --- | --- | --- | --- | --- | --- | --- | --- | --- | --- | --- | --- | --- | --- | --- | --- | --- | --- | --- | --- | --- | --- | --- | --- | --- | --- | --- |
|  | | | | | |  |  |  |  |  |  |  | | | |  |  |  |  |  |  |  | | | |  |  |  |  |  |
| **ROI-to-ROI** | |  |  | | |  |  |  | T | p_FDR_ | |  |  |  |  |  |  |  | T | p_FDR_ | |  |  |  |  |  |  |  | T | p_FDR_ |
|  | |  |  | | |  |  |  |  |  | |  |  |  |  |  |  |  |  |  | |  |  |  |  |  |  |  |  |  |
| *t-contrast: high WS ext > low WS ext* | | | | | | |  |  |  |  |  |  |  |  |  |  |  |  |  |  |  |  |  |  |  |  |  |  |  |  |
|  | | | | | |  |  |  |  |  |  |  |  |  |  |  |  |  |  |  |  |  |  |  |  |  |  |  |  |  |
| Superior frontal gyrus R ⟷ | | | | | Inferior frontal gyrus, pars triangularis L | | | | 4.36 | <.001 | | Superior frontal gyrus R ⟷ | | | | Inferior frontal gyrus, pars triangularis L | | | 4.42 | <.001 | | No differential connectivity | | | | | |  |  |  |
| Rectus R ⟷ | | | | | Inferior frontal gyrus, pars orbitalis L | | | | 3.23 | <.001 | | Rectus R ⟷ | | | | Inferior frontal gyrus, pars orbitalis L | | | 3.33 | <.05 | |  | | |  | | |  |  |  |
| Rectus L ⟷ | | | | | Inferior frontal gyrus, pars orbitalis L | | | | 3.02 | <.001 | | Rectus L ⟷ | | | | Inferior frontal gyrus, pars orbitalis L | | | 2.86 | <.05 | |  | | |  | | |  |  |  |
| Superior frontal gyrus L ⟷ | | | | | Inferior frontal gyrus, pars orbitalis L | | | | 3.17 | <.001 | | Superior frontal gyrus L ⟷ | | | | Inferior frontal gyrus, pars orbitalis L | | | 2.96 | <.05 | |  | | |  | | |  |  |  |
|  | | |  | | | | |  |  |  | |  | | |  | | |  |  |  | |  | | |  | | |  |  |  |
| *t-contrast: low WS ext > high WS ext* | | | | | | |  |  |  |  |  |  |  |  |  |  |  |  |  |  |  |  |  |  |  |  |  |  |  |  |
|  | | | | | |  |  |  |  |  |  |  |  |  |  |  |  |  |  |  |  |  |  |  |  |  |  |  |  |  |
| No differential connectivity | | | | | | | |  |  |  | | No differential connectivity | | | | | |  |  |  | | No differential connectivity | | | |  |  |  |  |  |
|  | | |  | | | | |  |  |  | |  | | |  | | |  |  |  | |  |  |  |  |  |  |  |  |  |
|  | |  |  | | |  |  |  |  |  | |  |  |  |  |  |  |  |  |  | |  |  |  |  |  |  |  |  |  |
| **Seed-to-Voxel** | | Side | k | | | x | y | z | T | p_FDR_ | |  |  | Side | k | x | y | z | T | p_FDR_ | |  |  | Side | k | x | y | z | T | p_FDR_ |
|  | |  |  | | |  |  |  |  |  |  |  | |  |  |  |  |  |  |  |  |  |  |  |  |  |  |  |  |  |
| Amygdala | |  |  | | |  |  |  |  |  |  |  | |  |  |  |  |  |  |  |  |  |  |  |  |  |  |  |  |  |
| *t-contrast: high WS ext > low WS ext* | | | | | | |  |  |  |  |  |  | | | |  |  |  |  |  |  |  |  |  |  |  |  |  |  |  |
|  | | | | | |  |  |  |  |  |  |  | | | |  |  |  |  |  |  |  |  |  |  |  |  |  |  |  |
| No differential connectivity | | | | | |  |  |  |  |  | | No differential connectivity | | | |  |  |  |  |  | | No differential connectivity | | | |  |  |  |  |  |
|  | |  |  | | |  |  |  |  |  |  |  | |  |  |  |  |  |  |  |  |  |  |  |  |  |  |  |  |  |
| *t-contrast: low WS ext > high WS ext* | | | | | | |  |  |  |  |  |  |  |  |  |  |  |  |  |  |  |  |  |  |  |  |  |  |  |  |
| *Cluster 1* |  | | | 166 | | 26 | 34 | 22 | 5.57 | <.001 | | *Cluster 1* | |  | 138 | -28 | 48 | 22 | 3.2 | <.01 | | No differential connectivity | | | |  |  |  |  |  |
| Middle frontal gyrus | R | | | 117 | |  |  |  |  |  |  | Middle frontal gyrus | |  |  |  |  |  |  |  |  |  | | | |  |  |  |  |  |
| unlabeled | -- | | | 49 | |  |  |  |  |  |  | Superior frontal gyrus | |  |  |  |  |  |  |  |  |  | | | |  |  |  |  |  |
|  |  | | |  | |  |  |  |  |  |  |  |  |  |  |  |  |  |  |  |  |  | | | |  |  |  |  |  |
| *Cluster 2* |  | | | 115 | | -28 | 48 | 22 | 4.64 | <.001 | |  |  |  |  |  |  |  |  |  |  |  | | | |  |  |  |  |  |
| Middle frontal gyrus | L | | | 109 | |  |  |  |  |  |  |  |  |  |  |  |  |  |  |  |  |  | | | |  |  |  |  |  |
| Superior frontal gyrus | L | | | 6 | |  |  |  |  |  |  |  |  |  |  |  |  |  |  |  |  |  | | | |  |  |  |  |  |

| *Table S2 to be continued.* | | | | |  |  |  |  |  |  |  |  |  |  |  |  |  |  |  |  |  |  |  |  |  |  |  |
| --- | --- | --- | --- | --- | --- | --- | --- | --- | --- | --- | --- | --- | --- | --- | --- | --- | --- | --- | --- | --- | --- | --- | --- | --- | --- | --- | --- |
| **Primary analyses** | | |  |  |  |  |  |  | **Cross-team replication** | | | |  |  |  |  |  |  | **Cross-site replication** | | | |  |  |  |  |  |
|  | | |  |  |  |  |  |  |  | | | |  |  |  |  |  |  |  | | | |  |  |  |  |  |
| **Seed-to-Voxel** | Side | k | x | y | z | T | p_FDR_ | |  |  | Side | k | x | y | z | T | p_FDR_ | |  |  | Side | k | x | y | z | T | p_FDR_ |
| Anterior cingulate cortex | | |  |  |  |  |  |  |  |  |  |  |  |  |  |  |  |  |  |  |  |  |  |  |  |  |  |
| *t-contrast: high WS ext > low WS ext* | | | |  |  |  |  |  |  |  |  |  |  |  |  |  |  |  |  |  |  |  |  |  |  |  |  |
|  | | | |  |  |  |  |  |  |  |  |  |  |  |  |  |  |  |  |  |  |  |  |  |  |  |  |
| No differential connectivity | | |  |  |  |  |  | | No differential connectivity | | | |  |  |  |  |  | | No differential connectivity | | | |  |  |  |  |  |
|  | | |  |  |  |  |  | |  | |  |  |  |  |  |  |  | |  | | | |  |  |  |  |  |
| *t-contrast: low WS ext > high WS ext* | | | |  |  |  |  |  |  |  |  |  |  |  |  |  |  |  |  |  |  |  |  |  |  |  |  |
|  | | | |  |  |  |  |  |  |  |  |  |  |  |  |  |  |  |  |  |  |  |  |  |  |  |  |
| No differential connectivity | | |  |  |  |  |  |  | No differential connectivity | | | |  |  |  |  |  |  | No differential connectivity | | | |  |  |  |  |  |
|  | | |  |  |  |  |  |  |  | | | |  |  |  |  |  |  |  | | | |  |  |  |  |  |
| Hippocampus | |  |  |  |  |  |  |  |  |  |  |  |  |  |  |  |  |  |  |  |  |  |  |  |  |  |  |
| *t-contrast: high WS ext > low WS ext* | | | |  |  |  |  |  |  |  |  |  |  |  |  |  |  |  |  |  |  |  |  |  |  |  |  |
|  | | |  |  |  |  |  |  |  |  |  |  |  |  |  |  |  |  |  |  |  |  |  |  |  |  |  |
| No differential connectivity | | |  |  |  |  |  | |  | |  |  |  |  |  |  |  | | *Cluster 1* | |  | 99 | 16 | -82 | 30 |  | <.05 |
|  |  |  |  |  |  |  |  | |  | |  |  |  |  |  |  |  |  | Occipital Superior cortex | | R | 81 | R |  |  |  |  |
|  |  |  |  |  |  |  |  | |  | |  |  |  |  |  |  |  |  | Cuneus | | R | 18 | R |  |  |  |  |
|  |  |  |  |  |  |  |  | |  | |  |  |  |  |  |  |  |  | Occipital Superior cortex | | R | 81 | R |  |  |  |  |
|  |  |  |  |  |  |  |  | |  | |  |  |  |  |  |  |  |  |  | |  |  |  |  |  |  |  |
|  |  |  |  |  |  |  |  | |  | |  |  |  |  |  |  |  |  | *Cluster 2* | |  | 93 | 14 | -64 | 2 |  | <.05 |
|  |  |  |  |  |  |  |  | |  | |  |  |  |  |  |  |  |  | Lingual Gyrus | | R | 80 |  |  |  |  |  |
|  |  |  |  |  |  |  |  | |  | |  |  |  |  |  |  |  |  | Calcarine | | R | 13 |  |  |  |  |  |
|  |  |  |  |  |  |  |  | |  | |  |  |  |  |  |  |  |  |  |  |  |  |  |  |  |  |  |
| *t-contrast: low WS ext > high WS ext* | | | |  |  |  |  |  |  |  |  |  |  |  |  |  |  |  |  |  |  |  |  |  |  |  |  |
|  | | |  |  |  |  |  |  |  |  |  |  |  |  |  |  |  |  |  |  |  |  |  |  |  |  |  |
| No differential connectivity | | |  |  |  |  |  |  |  | | | |  |  |  |  |  |  |  | | | |  |  |  |  |  |

*Notes:* FDR= False Discovery Rate; k= number of voxels per cluster/region; ROI= Region of Interest, SPQ= Spider Phobia Questionnaire; WS ext= Within-session fear extinction x, y, z, MNI coordinates.

Supplement 6

**Table S3.** Cross-team replication of seed-to-voxel analyses with slightly higher cluster threshold p< .06

| **Seed-to-Voxel** | Side | k | x | y | z | T | p_FDR_ | |
| --- | --- | --- | --- | --- | --- | --- | --- | --- |
| Amygdala | | |  |  |  |  |  |  |
| *t-contrast: resp > non-resp* | | |  |  |  |  |  |  |
| *Cluster 1:* |  | 137 | 6 | -86 | 6 | 3.2 | <.01 | |
| Calcarine | R | 88 |  |  |  |  |  |  |
| Lingual gyrus | R | 35 |  |  |  |  |  |  |
| Calcarine | L | 12 |  |  |  |  |  |  |
| Lingual Gyrus | L | 2 |  |  |  |  |  |  |
|  |  |  |  |  |  |  |  |  |
| *Cluster 2*: |  | 82 | -10 | -42 | 12 | 3.2 | <.06 |  |
| Cerebellum 4/5 |  | 64 |  |  |  |  |  | |
| Fusiform gyrus | L | 10 |  |  |  |  |  |  |
| Lingual gyrus | L | 6 |  |  |  |  |  |  |
| Cerebellum 3 | L | 2 |  |  |  |  |  |  |
|  |  |  |  |  |  |  |  |  |
| *Cluster 3:* |  | 78 | 16 | -52 | -12 | 3.2 | <.06 |  |
| Cerebellum 4/5 | R | 41 |  |  |  |  |  |  |
| Fusiform gyrus | R | 16 |  |  |  |  |  | |
| Lingual gyrus | R | 15 |  |  |  |  |  |  |
| Cerebellum 6 | R | 6 |  |  |  |  |  |  |

*Notes:* FDR= False Discovery Rate; k= number of voxels per cluster/region; non-resp= non-responder; resp= responder; x, y, z, MNI coordinates.

Supplement 7: Exploratory results of the combined sample

### 1 ROI-to-ROI approach

The exploratory comparison of SPQ-responders to SPQ-Non-responders, as well as BAT-responders/Non-responders and high vs. low WSF revealed no differential connectivity in the combined sample (see Supplement 7 Table S4).

### 2 Seed-to-Voxel approach

Amygdala

We did not find differential connectivity for the amygdala as see.

Anterior cingulate cortex

Seed-to-voxel analyses of the ACC revealed that the ACC was connected significantly more positive with left triangular IFG in patients exhibiting more anxiety reduction during exposure, T(142)= 3.36, *p*< 0.01, *d*= 0.55 (see Supplement 7 Table S4).

Hippocampus

Hippocampus was more positively connected to various clusters, i.e. two clusters in the pre- and post-central cortex, T(142)= 3.36, *p*< 0.001, *d*= 0.55 and a cluster predominantly located in the superior occipital cortex, T(142)= 3.36, *p*< 0.01, *d*= 0.55 (see Supplement 7 Table S4).

**Table S4***.* Differential functional resting-state connectivity in the combined sample (ROI-to-ROI and Seed-to-Voxel)

| **Analyses combined sample SPQ-responders and non-responders** |  |  |  |  |  |  |  |
| --- | --- | --- | --- | --- | --- | --- | --- |
|  |  |  |  |  |  |  |  |
| ROI-to ROI |  |  |  |  |  |  |  |
|  |  |  |  |  |  |  |  |
| *t-contrast: resp > non-resp* |  |  |  |  |  |  |  |
| No differential connectivity |  |  |  |  |  |  |  |
|  |  |  |  |  |  |  |  |
| **Seed-to-Voxel** | Side | k | x | y | z | T | p_FDR_ |
|  |  |  |  |  |  |  |  |
| Amygdala |  |  |  |  |  |  |  |
| t-contrast: resp > non-resp |  |  |  |  |  |  |  |
| No differential connectivity |  |  |  |  |  |  |  |
|  |  |  |  |  |  |  |  |
| *t-contrast: non-resp > resp* |  |  |  |  |  |  |  |
| No differential connectivity |  |  |  |  |  |  |  |
|  |  |  |  |  |  |  |  |
| Anterior cingulate cortex |  |  |  |  |  |  |  |
| *t-contrast: resp > non-resp* |  |  |  |  |  |  |  |
| No differential connectivity |  |  |  |  |  |  |  |
|  |  |  |  |  |  |  |  |
| *t-contrast: non-resp > resp* |  |  |  |  |  |  |  |
| No differential connectivity |  |  |  |  |  |  |  |
|  |  |  |  |  |  |  |  |
| Hippocampus |  |  |  |  |  |  |  |
| *t-contrast: resp > non-resp* |  |  |  |  |  |  |  |
| No differential connectivity |  |  |  |  |  |  |  |
|  |  |  |  |  |  |  |  |
| *t-contrast: non-resp > resp* |  |  |  |  |  |  |  |
| No differential connectivity |  |  |  |  |  |  |  |
|  |  |  |  |  |  |  |  |
| **Analyses combined sample BAT-responders and non-responders** |  |  |  |  |  |  |  |
|  |  |  |  |  |  |  |  |
| ROI-to ROI |  |  |  |  |  |  |  |
|  |  |  |  |  |  |  |  |
| *t-contrast: resp > non-resp* |  |  |  |  |  |  |  |
| No differential connectivity |  |  |  |  |  |  |  |
|  |  |  |  |  |  |  |  |
| **Seed-to-Voxel** | Side | k | x | y | z | T | p_FDR_ |
|  |  |  |  |  |  |  |  |
| Amygdala |  |  |  |  |  |  |  |
| t-contrast: resp > non-resp |  |  |  |  |  |  |  |
| No differential connectivity |  |  |  |  |  |  |  |
|  |  |  |  |  |  |  |  |
| *t-contrast: non-resp > resp* |  |  |  |  |  |  |  |
|  |  |  |  |  |  |  |  |
|  |  |  |  |  |  |  |  |
| Anterior cingulate cortex |  |  |  |  |  |  |  |
| *t-contrast: resp > non-resp* |  |  |  |  |  |  |  |
| No differential connectivity |  |  |  |  |  |  |  |
|  |  |  |  |  |  |  |  |
| *t-contrast: non-resp > resp* |  |  |  |  |  |  |  |
| No differential connectivity |  |  |  |  |  |  |  |
|  |  |  |  |  |  |  |  |
| Hippocampus |  |  |  |  |  |  |  |
| *t-contrast: resp > non-resp* |  |  |  |  |  |  |  |
| No differential connectivity |  |  |  |  |  |  |  |
|  |  |  |  |  |  |  |  |
| *t-contrast: non-resp > resp* |  |  |  |  |  |  |  |
| No differential connectivity |  |  |  |  |  |  |  |
|  |  |  |  |  |  |  |  |
| **Analyses combined sample high versus low within session fear reduction** | | |  |  |  |  |  |
|  |  |  |  |  |  |  |  |
| ROI-to ROI |  |  |  |  |  |  |  |
|  |  |  |  |  |  |  |  |
| *t-contrast: high WS ext > low WS ext* |  |  |  |  |  |  |  |
| No differential connectivity |  |  |  |  |  |  |  |
|  |  |  |  |  |  |  |  |
| **Seed-to-Voxel** | Side | k | x | y | z | T | p_FDR_ |
|  |  |  |  |  |  |  |  |
| Amygdala |  |  |  |  |  |  |  |
| t-contrast: *high WS ext > low WS ext* |  |  |  |  |  |  |  |
| No differential connectivity |  |  |  |  |  |  |  |
|  |  |  |  |  |  |  |  |
| *t-contrast: low WS ext > high WS ext* |  |  |  |  |  |  |  |
| No differential connectivity |  |  |  |  |  |  |  |
|  |  |  |  |  |  |  |  |
| Anterior cingulate cortex |  |  |  |  |  |  |  |
| *t-contrast: high WS ext > low WS ext* |  |  |  |  |  |  |  |
| Cluster 1 | L | 188 | -52 | 20 | 30 | 3.36 | <.01 |
| Fonrtal Inferior Cortex, part. Tri | L | 186 |  |  |  |  |  |
| Frontal Mid | L | 2 |  |  |  |  |  |
|  |  |  |  |  |  |  |  |
| *t-contrast: low WS ext > high WS ext* |  |  |  |  |  |  |  |
| Same results as in *high WS ext > low WS ext* but negative connectivity |  |  |  |  |  |  |  |
|  |  |  |  |  |  |  |  |
| Hippocampus |  |  |  |  |  |  |  |
| *t-contrast: high WS ext > low WS ext* |  |  |  |  |  |  |  |
| Cluster 1 | R | 202 | 42 | -18 | 62 | 3.36 | <.0001 |
| Precentral Cortex | R | 127 |  |  |  |  |  |
| Postecentral Cortex | R | 74 |  |  |  |  |  |
| not-labeled |  | 1 |  |  |  |  |  |
|  |  |  |  |  |  |  |  |
| Cluster 2 | L | 109 | -20 | -72 | 36 |  | <.05 |
| Occipital superior | L | 89 |  |  |  |  |  |
| Cuneus | L | 13 |  |  |  |  |  |
| Parietal superior | L | 3 |  |  |  |  |  |
| Precuneus | L | 3 |  |  |  |  |  |
| not-labeled |  | 1 |  |  |  |  |  |
|  |  |  |  |  |  |  |  |
| Cluster 3 | L | 107 | -42 | -20 | 42 |  | <.05 |
| Postcentral | L | 91 |  |  |  |  |  |
| Precentral | L | 13 |  |  |  |  |  |
| Parietal inferior | L | 3 |  |  |  |  |  |
|  |  |  |  |  |  |  |  |
| *t-contrast: low WS ext > high WS ext* |  |  |  |  |  |  |  |
| Same results as in *high WS ext > low WS ext* but inhibitory |  |  |  |  |  |  |  |
|  |  |  |  |  |  |  |  |

*Notes:* FDR= False Discovery Rate; k= number of voxels per cluster/region; non-resp= Non-Responder; resp= responder; ROI, Region of Interest, SPQ= Spider Phobia Questionnaire; BAT= Behavioral Avoidance Test; x, y, z, MNI coordinates.”
